# Ethyl 1-formamido-4-oxo-2,6-diphenyl­cyclo­hexa­necarboxyl­ate

**DOI:** 10.1107/S1600536811000985

**Published:** 2011-01-15

**Authors:** Dawei Zhang, Xianxiu Xu, Qun Liu

**Affiliations:** a Department of Chemistry, Yanbian University, Yanji 133002, People’s Republic of China; b Department of Chemistry, Northeast Normal University, Changchun 130024, People’s Republic of China

## Abstract

In the title compound, C_22_H_23_NO_4_, the central six-membered ring is in a twist-boat conformation, the two aryl groups are in equatorial positions and the dihedral angle between the two aromatic rings is 75.98 (12)°.

## Related literature

For the synthesis, see: Tan *et al.* (2009 ▶); Zhang *et al.* (2010 ▶). For related structures, see: Rowland & Gill (1988 ▶); Rowland *et al.* (1998 ▶); Aleman *et al.* (2009 ▶). Cyclic constrained analogues of phenyl­alanine (Phe) are of particular inter­est in the construction of peptide analogues with controlled folds in the backbone because they play an important role in both restricting the χ_1_ torsion angle and in peptide receptor recognition processes, see: Cativiela & Díaz-de-Villegas (1998 ▶, 2000 ▶, 2007 ▶); Cativiela & Ordóñez (2009 ▶); Lasa & Cativiela (2006 ▶).
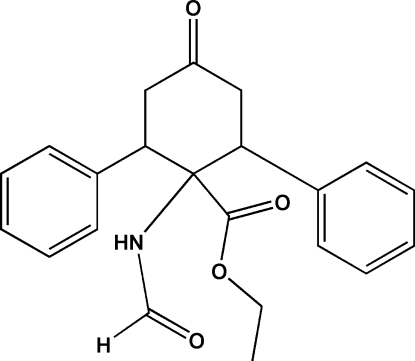


## Experimental

### 

#### Crystal data

C_22_H_23_NO_4_
*M* *_r_* = 365.41
Monoclinic, 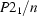

*a* = 11.3240 (12) Å
*b* = 13.5100 (15) Å
*c* = 12.5870 (14) Å
β = 99.149 (2)°
*V* = 1901.2 (4) Å^3^
*Z* = 4
Mo *K*α radiation
μ = 0.09 mm^−1^
*T* = 293 K
0.21 × 0.16 × 0.14 mm

#### Data collection

Bruker SMART APEXII CCD area-detector diffractometer
Absorption correction: multi-scan (*SADABS*; Sheldrick, 1996 ▶) *T*_min_ = 0.982, *T*_max_ = 0.988
11361 measured reflections
4439 independent reflections
3120 reflections with *I* > 2σ(*I*)
*R*_int_ = 0.021

#### Refinement

*R*[*F* ^2^ > 2σ(*F* ^2^)] = 0.042
*wR*(*F* ^2^) = 0.116
*S* = 1.02
4439 reflections
244 parameters
H-atom parameters constrained
Δρ_max_ = 0.22 e Å^−3^
Δρ_min_ = −0.19 e Å^−3^

### 

Data collection: *APEX2* (Bruker, 2007 ▶); cell refinement: *SAINT* (Bruker, 2007 ▶); data reduction: *SAINT*; program(s) used to solve structure: *SHELXS97* (Sheldrick, 2008 ▶); program(s) used to refine structure: *SHELXL97* (Sheldrick, 2008 ▶); molecular graphics: *SHELXTL* (Sheldrick, 2008 ▶); software used to prepare material for publication: *SHELXTL*.

## Supplementary Material

Crystal structure: contains datablocks I, global. DOI: 10.1107/S1600536811000985/nc2214sup1.cif

Structure factors: contains datablocks I. DOI: 10.1107/S1600536811000985/nc2214Isup2.hkl

Additional supplementary materials:  crystallographic information; 3D view; checkCIF report

## Figures and Tables

**Fig. 1.**
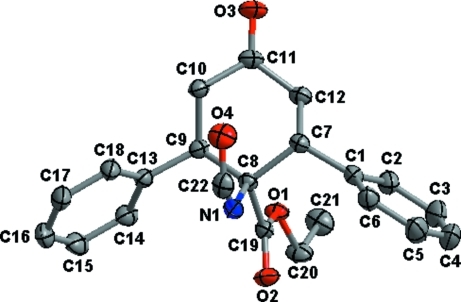
View of the molecular structure of the title compound with labeling and displacement ellipsoids drawn at the 30% probability level.

## supplementary crystallographic information

### Comment

The cyclic constrained analogues of phenylalanine (Phe) are of particular interest in the construction of peptide analogues with controlled fold in the backbone, because they play an important role in both restricting torsional angle χ1 and peptide receptor recognition processes (Cativiela *et al.* 1998; Cativiela *et al.* 2000. Cativiela *et al.* 2007; Cativiela *et al.* 2009; Lasa *et al.* 2006). The crystal structure of title compound, a phenyl substituted highly constrained cyclohexane analogue of Phe, is reported in this paper.

In the crystal structure the central six-membered ring is in a twist conformation which can presumably traced back due to steric hindrance of the ethoxyl carbonyl, the amide and the two aryl groups (Fig. 1). The two aryl groups are located in equatorial positions and the dihedral angle between two aromatic rings amount to 75.98 (12)°.

### Experimental

To a mixture of (1E,4E)-1,5-bis(4-chlorophenyl)penta-1,4-dien-3-one (303 mg, 1.0 mmol) and ethyl isocyanoacetate (0.132 ml, 1.2 mmol) in DMF (5 ml) was added 1,8-diazabicyclo [5.4.0]undec-7-ene (DBU) (0.015 ml, 0.1 mmol) in one portion at room temperature. The reaction mixture was stirre at room temperature, and the reaction mixture was monitored by TLC. After the substrate (1E,4E)-1,5-bis(4-chlorophenyl)penta-1,4-dien-3-one was consumed, the resulting mixture was poured into ice-water (30 ml) under stirring. The precipitate was cfiltered off, washed with water (3 × 10 ml), and dried *in vacuo* to afford the crude product ethyl 2,6-bis(4-chlorophenyl)-1-isocyano-4-oxocyclohexanecarboxylate, which was purified by flash chromatography (silica gel, petroleum ether: diethyl ether = 3: 1, V/V) to give ethyl 2,6-bis(4-chlorophenyl)-1-isocyano-4-oxocyclohexanecarboxylate (387 mg, 93%). Colorless single crystals of the title compound were obtained by slow evaporation of the sovent from an ethanol solution at room temperature.

### Refinement

The N-bound H atom was located in a difference map, fixed at this position and refined as riding with *U*iso(H) = 1.2*U*eq(N). The remaining hydrogen atoms were placed in ideal positions (C—H = 0.93–0.98 Å) and refined as riding with *U*iso(H) = 1.2*U*eq(C) or 1.5*U*eq(methyl C). The methyl groups were allowed to rotate, but not to tip.

### Crystal data

**Table:**

C_22_H_23_NO_4_	*F*(000) = 776
*M**_r_* = 365.41	*D*_x_ = 1.277 Mg m^−^^3^
Monoclinic, *P*2_1_/*n*	Mo *K*α radiation, λ = 0.71069 Å
*a* = 11.3240 (12) Å	Cell parameters from 132 reflections
*b* = 13.5100 (15) Å	θ = 1.3–26.0°
*c* = 12.5870 (14) Å	µ = 0.09 mm^−^^1^
β = 99.149 (2)°	*T* = 293 K
*V* = 1901.2 (4) Å^3^	Block, colorless
*Z* = 4	0.21 × 0.16 × 0.14 mm

### Data collection

**Table:**

Bruker SMART APEXII CCD area-detector diffractometer	4439 independent reflections
Radiation source: fine-focus sealed tube	3120 reflections with *I* > 2σ(*I*)
graphite	*R*_int_ = 0.021
ω scans	θ_max_ = 28.3°, θ_min_ = 2.2°
Absorption correction: multi-scan (*SADABS*; Sheldrick, 1996)	*h* = −15→14
*T*_min_ = 0.982, *T*_max_ = 0.988	*k* = −16→17
11361 measured reflections	*l* = −14→16

### Refinement

**Table:**

Refinement on *F*^2^	Primary atom site location: structure-invariant direct methods
Least-squares matrix: full	Secondary atom site location: difference Fourier map
*R*[*F*^2^ > 2σ(*F*^2^)] = 0.042	Hydrogen site location: inferred from neighbouring sites
*wR*(*F*^2^) = 0.116	H-atom parameters constrained
*S* = 1.02	*w* = 1/[σ^2^(*F*_o_^2^) + (0.0534*P*)^2^ + 0.306*P*] where *P* = (*F*_o_^2^ + 2*F*_c_^2^)/3
4439 reflections	(Δ/σ)_max_ < 0.001
244 parameters	Δρ_max_ = 0.22 e Å^−^^3^
0 restraints	Δρ_min_ = −0.19 e Å^−^^3^

### Special details

**Table:**

Geometry. All e.s.d.'s (except the e.s.d. in the dihedral angle between two l.s. planes) are estimated using the full covariance matrix. The cell e.s.d.'s are taken into account individually in the estimation of e.s.d.'s in distances, angles and torsion angles; correlations between e.s.d.'s in cell parameters are only used when they are defined by crystal symmetry. An approximate (isotropic) treatment of cell e.s.d.'s is used for estimating e.s.d.'s involving l.s. planes.
Refinement. Refinement of *F*^2^ against ALL reflections. The weighted *R*-factor *wR* and goodness of fit *S* are based on *F*^2^, conventional *R*-factors *R* are based on *F*, with *F* set to zero for negative *F*^2^. The threshold expression of *F*^2^ > σ(*F*^2^) is used only for calculating *R*-factors(gt) *etc*. and is not relevant to the choice of reflections for refinement. *R*-factors based on *F*^2^ are statistically about twice as large as those based on *F*, and *R*- factors based on ALL data will be even larger.

### Fractional atomic coordinates and isotropic or equivalent isotropic displacement parameters (Å^2^)

**Table:**

	*x*	*y*	*z*	*U*_iso_*/*U*_eq_	
O2	1.03050 (10)	0.14890 (8)	0.47576 (8)	0.0541 (3)	
O1	0.94334 (8)	0.26361 (7)	0.36180 (7)	0.0426 (2)	
C19	0.98394 (11)	0.17327 (10)	0.38744 (10)	0.0364 (3)	
N1	1.01302 (10)	0.00919 (8)	0.33120 (9)	0.0401 (3)	
C8	0.95919 (11)	0.10312 (9)	0.29070 (10)	0.0339 (3)	
C1	0.75462 (12)	0.08726 (11)	0.35380 (11)	0.0416 (3)	
O4	0.95020 (11)	−0.09967 (8)	0.19437 (10)	0.0637 (3)	
O3	0.76178 (11)	0.15572 (10)	−0.01954 (9)	0.0699 (4)	
C7	0.81977 (11)	0.09052 (10)	0.25754 (10)	0.0366 (3)	
H7	0.8083	0.0251	0.2239	0.044*	
C10	0.94777 (13)	0.11160 (11)	0.08583 (11)	0.0443 (3)	
H10A	0.9870	0.1368	0.0283	0.053*	
H10B	0.9497	0.0399	0.0832	0.053*	
C13	1.14993 (12)	0.13184 (11)	0.20876 (11)	0.0408 (3)	
C9	1.01555 (12)	0.14705 (10)	0.19429 (10)	0.0378 (3)	
H9	1.0031	0.2188	0.1961	0.045*	
C12	0.76820 (13)	0.16456 (11)	0.17030 (11)	0.0448 (3)	
H12A	0.6819	0.1579	0.1554	0.054*	
H12B	0.7870	0.2315	0.1955	0.054*	
C20	0.94851 (15)	0.33379 (11)	0.45037 (13)	0.0534 (4)	
H20A	1.0308	0.3516	0.4773	0.064*	
H20B	0.9137	0.3050	0.5088	0.064*	
C11	0.81977 (13)	0.14617 (11)	0.06898 (11)	0.0456 (3)	
C18	1.20075 (13)	0.04802 (12)	0.17129 (12)	0.0510 (4)	
H18	1.1518	0.0000	0.1342	0.061*	
C14	1.22578 (14)	0.20301 (12)	0.26263 (12)	0.0496 (4)	
H14	1.1936	0.2601	0.2878	0.060*	
C2	0.70022 (14)	0.16996 (13)	0.38990 (13)	0.0550 (4)	
H2	0.7006	0.2295	0.3528	0.066*	
C6	0.74950 (14)	−0.00068 (14)	0.40968 (14)	0.0563 (4)	
H6	0.7837	−0.0576	0.3861	0.068*	
C22	1.00363 (14)	−0.07956 (11)	0.28331 (14)	0.0500 (4)	
H22	1.0424	−0.1318	0.3225	0.060*	
C15	1.34882 (15)	0.18981 (14)	0.27916 (14)	0.0610 (5)	
H15	1.3985	0.2380	0.3152	0.073*	
C17	1.32359 (15)	0.03502 (15)	0.18842 (14)	0.0621 (5)	
H17	1.3564	−0.0218	0.1633	0.075*	
C16	1.39736 (15)	0.10596 (16)	0.24257 (14)	0.0648 (5)	
H16	1.4798	0.0970	0.2542	0.078*	
C3	0.64542 (17)	0.16480 (18)	0.48052 (17)	0.0773 (6)	
H3	0.6105	0.2212	0.5044	0.093*	
C5	0.69387 (18)	−0.00491 (19)	0.50051 (17)	0.0799 (6)	
H5	0.6919	−0.0642	0.5378	0.096*	
C21	0.87950 (17)	0.42237 (12)	0.40695 (16)	0.0659 (5)	
H21A	0.8803	0.4706	0.4631	0.099*	
H21B	0.7984	0.4036	0.3803	0.099*	
H21C	0.9151	0.4502	0.3494	0.099*	
C4	0.64203 (19)	0.0781 (2)	0.53508 (17)	0.0882 (7)	
H4	0.6046	0.0753	0.5957	0.106*	
H1N	1.0524	0.0128	0.3939	0.106*	

### Atomic displacement parameters (Å^2^)

**Table:**

	*U*^11^	*U*^22^	*U*^33^	*U*^12^	*U*^13^	*U*^23^
O2	0.0651 (7)	0.0600 (7)	0.0331 (5)	0.0161 (5)	−0.0045 (5)	−0.0018 (5)
O1	0.0489 (6)	0.0382 (5)	0.0375 (5)	0.0043 (4)	−0.0026 (4)	−0.0064 (4)
C19	0.0337 (6)	0.0409 (7)	0.0339 (7)	0.0032 (5)	0.0027 (5)	−0.0003 (5)
N1	0.0411 (6)	0.0377 (6)	0.0415 (6)	0.0070 (5)	0.0065 (5)	0.0038 (5)
C8	0.0345 (6)	0.0335 (6)	0.0333 (7)	0.0026 (5)	0.0039 (5)	0.0012 (5)
C1	0.0298 (6)	0.0528 (8)	0.0411 (8)	0.0000 (6)	0.0021 (5)	0.0003 (6)
O4	0.0742 (8)	0.0462 (6)	0.0687 (8)	0.0036 (5)	0.0055 (6)	−0.0131 (6)
O3	0.0697 (8)	0.0954 (9)	0.0387 (6)	0.0093 (7)	−0.0093 (5)	0.0077 (6)
C7	0.0346 (7)	0.0372 (7)	0.0367 (7)	−0.0003 (5)	0.0015 (5)	−0.0011 (5)
C10	0.0484 (8)	0.0517 (8)	0.0321 (7)	−0.0063 (6)	0.0044 (6)	−0.0016 (6)
C13	0.0413 (7)	0.0501 (8)	0.0318 (7)	−0.0082 (6)	0.0078 (5)	0.0002 (6)
C9	0.0419 (7)	0.0388 (7)	0.0323 (7)	−0.0032 (6)	0.0052 (5)	−0.0006 (5)
C12	0.0406 (7)	0.0484 (8)	0.0424 (8)	0.0039 (6)	−0.0030 (6)	0.0016 (6)
C20	0.0587 (9)	0.0488 (9)	0.0482 (9)	0.0065 (7)	−0.0051 (7)	−0.0169 (7)
C11	0.0518 (8)	0.0444 (8)	0.0374 (8)	−0.0036 (6)	−0.0032 (6)	0.0040 (6)
C18	0.0436 (8)	0.0646 (10)	0.0463 (8)	−0.0092 (7)	0.0120 (7)	−0.0124 (7)
C14	0.0524 (9)	0.0514 (9)	0.0445 (8)	−0.0142 (7)	0.0065 (7)	−0.0011 (7)
C2	0.0445 (8)	0.0649 (10)	0.0566 (10)	0.0045 (7)	0.0109 (7)	−0.0073 (8)
C6	0.0443 (8)	0.0663 (10)	0.0599 (10)	0.0038 (7)	0.0127 (7)	0.0158 (8)
C22	0.0538 (9)	0.0384 (8)	0.0602 (10)	0.0095 (6)	0.0166 (8)	0.0050 (7)
C15	0.0517 (9)	0.0753 (12)	0.0542 (10)	−0.0266 (9)	0.0026 (8)	0.0007 (9)
C17	0.0460 (9)	0.0851 (13)	0.0588 (10)	−0.0002 (8)	0.0195 (8)	−0.0123 (9)
C16	0.0389 (8)	0.0963 (14)	0.0615 (11)	−0.0126 (9)	0.0150 (8)	−0.0043 (10)
C3	0.0604 (11)	0.1077 (17)	0.0673 (12)	0.0159 (11)	0.0212 (9)	−0.0169 (12)
C5	0.0580 (11)	0.1129 (17)	0.0710 (13)	0.0032 (11)	0.0172 (10)	0.0370 (12)
C21	0.0682 (11)	0.0490 (10)	0.0774 (12)	0.0083 (8)	0.0022 (9)	−0.0117 (8)
C4	0.0616 (12)	0.148 (2)	0.0601 (12)	0.0153 (13)	0.0267 (10)	0.0114 (14)

### Geometric parameters (Å, °)

**Table:**

O2—C19	1.1983 (15)	C12—H12B	0.9700
O1—C19	1.3258 (16)	C20—C21	1.485 (2)
O1—C20	1.4575 (16)	C20—H20A	0.9700
C19—C8	1.5333 (18)	C20—H20B	0.9700
N1—C22	1.3386 (19)	C18—C17	1.384 (2)
N1—C8	1.4639 (16)	C18—H18	0.9300
N1—H1N	0.8440	C14—C15	1.387 (2)
C8—C9	1.5744 (18)	C14—H14	0.9300
C8—C7	1.5768 (17)	C2—C3	1.384 (3)
C1—C6	1.387 (2)	C2—H2	0.9300
C1—C2	1.387 (2)	C6—C5	1.391 (3)
C1—C7	1.5161 (19)	C6—H6	0.9300
O4—C22	1.2157 (19)	C22—H22	0.9300
O3—C11	1.2070 (17)	C15—C16	1.370 (3)
C7—C12	1.5308 (18)	C15—H15	0.9300
C7—H7	0.9800	C17—C16	1.378 (2)
C10—C11	1.505 (2)	C17—H17	0.9300
C10—C9	1.5326 (18)	C16—H16	0.9300
C10—H10A	0.9700	C3—C4	1.361 (3)
C10—H10B	0.9700	C3—H3	0.9300
C13—C18	1.386 (2)	C5—C4	1.369 (3)
C13—C14	1.392 (2)	C5—H5	0.9300
C13—C9	1.5176 (19)	C21—H21A	0.9600
C9—H9	0.9800	C21—H21B	0.9600
C12—C11	1.505 (2)	C21—H21C	0.9600
C12—H12A	0.9700	C4—H4	0.9300
			
C19—O1—C20	116.26 (10)	O1—C20—H20B	110.4
O2—C19—O1	124.27 (12)	C21—C20—H20B	110.4
O2—C19—C8	124.39 (12)	H20A—C20—H20B	108.6
O1—C19—C8	111.30 (10)	O3—C11—C12	122.48 (14)
C22—N1—C8	128.38 (12)	O3—C11—C10	122.25 (14)
C22—N1—H1N	117.8	C12—C11—C10	115.24 (12)
C8—N1—H1N	113.8	C17—C18—C13	120.78 (15)
N1—C8—C19	104.24 (10)	C17—C18—H18	119.6
N1—C8—C9	113.44 (11)	C13—C18—H18	119.6
C19—C8—C9	109.50 (10)	C15—C14—C13	120.78 (16)
N1—C8—C7	110.03 (10)	C15—C14—H14	119.6
C19—C8—C7	109.02 (10)	C13—C14—H14	119.6
C9—C8—C7	110.37 (10)	C3—C2—C1	120.67 (18)
C6—C1—C2	117.88 (15)	C3—C2—H2	119.7
C6—C1—C7	119.68 (13)	C1—C2—H2	119.7
C2—C1—C7	122.44 (13)	C1—C6—C5	120.85 (18)
C1—C7—C12	114.43 (11)	C1—C6—H6	119.6
C1—C7—C8	112.68 (10)	C5—C6—H6	119.6
C12—C7—C8	111.78 (11)	O4—C22—N1	127.38 (14)
C1—C7—H7	105.7	O4—C22—H22	116.3
C12—C7—H7	105.7	N1—C22—H22	116.3
C8—C7—H7	105.7	C16—C15—C14	120.19 (15)
C11—C10—C9	111.29 (12)	C16—C15—H15	119.9
C11—C10—H10A	109.4	C14—C15—H15	119.9
C9—C10—H10A	109.4	C16—C17—C18	120.25 (17)
C11—C10—H10B	109.4	C16—C17—H17	119.9
C9—C10—H10B	109.4	C18—C17—H17	119.9
H10A—C10—H10B	108.0	C15—C16—C17	119.80 (16)
C18—C13—C14	118.20 (14)	C15—C16—H16	120.1
C18—C13—C9	122.16 (12)	C17—C16—H16	120.1
C14—C13—C9	119.63 (13)	C4—C3—C2	120.76 (19)
C13—C9—C10	114.53 (11)	C4—C3—H3	119.6
C13—C9—C8	112.22 (11)	C2—C3—H3	119.6
C10—C9—C8	111.21 (11)	C4—C5—C6	120.05 (19)
C13—C9—H9	106.1	C4—C5—H5	120.0
C10—C9—H9	106.1	C6—C5—H5	120.0
C8—C9—H9	106.1	C20—C21—H21A	109.5
C11—C12—C7	110.16 (12)	C20—C21—H21B	109.5
C11—C12—H12A	109.6	H21A—C21—H21B	109.5
C7—C12—H12A	109.6	C20—C21—H21C	109.5
C11—C12—H12B	109.6	H21A—C21—H21C	109.5
C7—C12—H12B	109.6	H21B—C21—H21C	109.5
H12A—C12—H12B	108.1	C3—C4—C5	119.78 (19)
O1—C20—C21	106.74 (12)	C3—C4—H4	120.1
O1—C20—H20A	110.4	C5—C4—H4	120.1
C21—C20—H20A	110.4		
			
C20—O1—C19—O2	−5.0 (2)	C7—C8—C9—C13	−162.62 (11)
C20—O1—C19—C8	172.73 (12)	N1—C8—C9—C10	91.15 (13)
C22—N1—C8—C19	170.75 (13)	C19—C8—C9—C10	−152.88 (11)
C22—N1—C8—C9	−70.21 (17)	C7—C8—C9—C10	−32.86 (15)
C22—N1—C8—C7	53.98 (18)	C1—C7—C12—C11	−167.17 (11)
O2—C19—C8—N1	−4.36 (18)	C8—C7—C12—C11	63.22 (14)
O1—C19—C8—N1	177.89 (10)	C19—O1—C20—C21	−169.98 (13)
O2—C19—C8—C9	−126.04 (14)	C7—C12—C11—O3	143.48 (15)
O1—C19—C8—C9	56.21 (14)	C7—C12—C11—C10	−34.51 (16)
O2—C19—C8—C7	113.11 (14)	C9—C10—C11—O3	155.50 (14)
O1—C19—C8—C7	−64.64 (14)	C9—C10—C11—C12	−26.50 (17)
C6—C1—C7—C12	148.86 (13)	C14—C13—C18—C17	0.9 (2)
C2—C1—C7—C12	−31.95 (18)	C9—C13—C18—C17	−178.16 (15)
C6—C1—C7—C8	−81.98 (16)	C18—C13—C14—C15	−0.6 (2)
C2—C1—C7—C8	97.21 (15)	C9—C13—C14—C15	178.47 (14)
N1—C8—C7—C1	76.19 (13)	C6—C1—C2—C3	1.6 (2)
C19—C8—C7—C1	−37.55 (14)	C7—C1—C2—C3	−177.59 (14)
C9—C8—C7—C1	−157.86 (11)	C2—C1—C6—C5	−1.5 (2)
N1—C8—C7—C12	−153.29 (11)	C7—C1—C6—C5	177.76 (15)
C19—C8—C7—C12	92.97 (13)	C8—N1—C22—O4	2.0 (3)
C9—C8—C7—C12	−27.35 (14)	C13—C14—C15—C16	−0.1 (2)
C18—C13—C9—C10	−38.46 (19)	C13—C18—C17—C16	−0.5 (3)
C14—C13—C9—C10	142.52 (14)	C14—C15—C16—C17	0.5 (3)
C18—C13—C9—C8	89.57 (16)	C18—C17—C16—C15	−0.3 (3)
C14—C13—C9—C8	−89.45 (15)	C1—C2—C3—C4	−1.1 (3)
C11—C10—C9—C13	−169.26 (12)	C1—C6—C5—C4	0.8 (3)
C11—C10—C9—C8	62.20 (15)	C2—C3—C4—C5	0.4 (3)
N1—C8—C9—C13	−38.62 (15)	C6—C5—C4—C3	−0.2 (3)
C19—C8—C9—C13	77.35 (13)		
